# The impact of hematocrit on oxygenation-sensitive cardiovascular magnetic resonance

**DOI:** 10.1186/s12968-016-0262-1

**Published:** 2016-07-20

**Authors:** Dominik P. Guensch, Gobinath Nadeshalingam, Kady Fischer, Aurelien F. Stalder, Matthias G. Friedrich

**Affiliations:** Philippa & Marvin Carsley CMR Centre at the Montreal Heart Institute, Montreal, QC Canada; Department of Anesthesiology and Pain Therapy, Inselspital, Bern University Hospital, University of Bern, Freiburgstrasse, 3010 Bern, Switzerland; Instutite of Diagnostic, Interventional and Pediatric Radiology, Inselspital, Bern University Hospital, University of Bern, Bern, Switzerland; Siemens Healthcare GmbH, Erlangen, Germany; Department of Medicine, Heidelberg University, Heidelberg, Germany; Departments of Cardiac Sciences and Radiology, University of Calgary, Calgary, AB Canada; Department of Radiology, Université de Montréal, Montreal, QC Canada; Departments of Medicine and Radiology, McGill University Health Centre, Montreal, QC Canada

**Keywords:** Hematocrit, Hemoglobin, Hemodilution, BOLD-CMR, OS-CMR, T2*

## Abstract

**Background:**

Oxygenation-sensitive (OS) Cardiovascular Magnetic Resonance (CMR) is a promising utility in the diagnosis of heart disease. Contrast in OS-CMR images is generated through deoxyhemoglobin in the tissue, which is negatively correlated with the signal intensity (SI). Thus, changing hematocrit levels may be a confounder in the interpretation of OS-CMR results. We hypothesized that hemodilution confounds the observed signal intensity in OS-CMR images.

**Methods:**

Venous and arterial blood from five pigs was diluted with lactated Ringer solution in 10 % increments to 50 %. The changes in signal intensity (SI) were compared to changes in blood gases and hemoglobin concentration. We performed an OS-CMR scan in 21 healthy volunteers using vasoactive breathing stimuli at baseline, which was then repeated after rapid infusion of 1 L of lactated Ringer’s solution within 5–8 min. Changes of SI were measured and compared between the hydration states.

**Results:**

The % change in SI from baseline for arterial (*r* = -0.67, *p* < 0.0001) and venous blood (*r* = -0.55, *p* = 0.002) were negatively correlated with the changes in hemoglobin (Hb). SI changes in venous blood were also associated with SO_2_ (*r* = 0.68, *p* < 0.0001) and deoxyHb concentration (-0.65, *p* < 0.0001). In healthy volunteers, rapid infusion resulted in a significant drop in the hemoglobin concentration (142.5 ± 15.2 g/L vs. 128.8 ± 15.2 g/L; *p* < 0.0001). Baseline myocardial SI increased by 3.0 ± 5.7 % (*p* = 0.026) following rapid infusion, and in males there was a strong association between the change in hemoglobin concentration and % changes in SI (*r* = 0.82, *p* = 0.002). After hyperhydration, the SI response after hyperventilation was attenuated (HV, *p* = 0.037), as was the maximum SI increase during apnea (*p* = 0.012). The extent of SI attenuation was correlated with the reduction in hemoglobin concentration at the end of apnea (*r* = 0.55, *p* = 0.012) for all subjects and at maximal SI (*r* = 0.63, *p* = 0.037) and the end of breath-hold (*r* = 0.68, *p* = 0.016) for males only.

**Conclusion:**

In dynamic studies using oxygenation-sensitive CMR, the hematocrit level affects baseline signal intensity and the observed signal intensity response. Thus, the hydration status of the patient may be a confounder for OS-CMR image analysis.

## Background

Oxygenation-sensitive (OS) Cardiovascular Magnetic Resonance (CMR) is a promising tool to assess myocardial oxygenation changes [1]. In contrast to other imaging modalities like coronary angiography or cardiac CT, it is not an anatomy-derived surrogate parameter for myocardial perfusion and oxygen supply, as OS-CMR detects net changes in myocardial oxygenation incorporating the contributing factors like blood oxygenation, blood flow, myocardial workload and oxygen extraction but also collateralization or coronary steal [2]. OS-CMR is based on the so-called Blood Oxygen Level-Dependent (BOLD) effect, which takes advantage of the paramagnetic deoxygenated hemoglobin as an inherent contrast. An increase in deoxyhemoglobin would result in a decrease in signal intensity in T2*-weighted sequences. Hence, a decrease in tissue oxygenation corresponds to a decrease in signal intensity. This technique was first proposed by Ogawa et al. and has since been used in functional MRI (fMRI) studies [3], which detects the activation of brain areas in response to distinct stimuli or triggered by tasks with an increase in local blood flow [4]. In recent cardiac studies, this technique has been used together with coronary vasodilators such as adenosine to measure the oxygenation reserve, by comparing the images under adenosine to a set of baseline images. Myocardial segments that fail to show an increase have a high probability of being subtended to a relevant coronary artery stenosis, being already maximally dilated after a fixed obstruction and thus failing to respond to adenosine [5–8]. With newer sequences, the temporal and spatial resolution is sufficient to use OS-CMR to detect rapid and dynamic myocardial oxygenation changes using breathing maneuvers. Breathing maneuvers such as hyperventilation and apnea result in systemic changes in blood carbon dioxide levels, which act as a strong modifier of coronary vascular tone [9, 10]. The sequence of hyperventilation with subsequent apnea has shown to yield changes in myocardial oxygenation at least as strong as adenosine infusion in healthy volunteers [11]. Such a protocol would not only be free of radiation and pharmacologic contrast agents, but would also require neither potentially dangerous vasodilators nor uncomfortable carbon dioxide levels adjusted by complicated and expensive breathing circuits [12]. OS-CMR is being utilized increasingly to investigate cardiac pathologies and offers an innovative and potentially clinically feasible diagnostic protocol together with breathing maneuvers [1, 2, 11, 13–15].

However, many factors influencing the technical aspects of the underlying T2*-sensitive sequences remain confounders. The hydration status of an individual may be such a confounder [16]. Lin et al. (1998) could show that hemodilution has an effect on baseline signal intensity in fMRI brain scans that use the same sequence principle [17, 18]. More importantly, hemodilution has been shown to influence the relative signal response during the execution of identical tasks in fMRI scans [19, 20].

The aim of this study was to assess the differences in oxygenation-sensitive signal intensity free of vasoactive effects in an in-vitro setup and during vasoactive breathing maneuvers in healthy subjects. We hypothesized that hemodilution results in an increase in baseline signal and leads to an attenuation in signal intensity changes after hemodilution during dynamic breathing maneuvers.

## Methods

### In-vitro study

Venous and fully oxygenated arterial blood samples from five pigs ventilated with 100 % oxygen were used to assess hemoglobin-dependent changes in oxygenation-sensitive (OS) signal intensity (SI) independent of vasomotor effects. The blood was obtained immediately after euthanasia of five animals used for another experiment [21]. The study was conducted in accordance with the Guide to the Care and Use of Experimental Animals by the Canadian Council on Animal Care and approved by the local Animal Care and Use Board and followed internationally accepted guidelines. The arterial (A100) and venous (V100) blood was diluted with Lactated Ringer’s solution in 10 % steps down to 50 % of the original sample (A50 and V50, respectively), and stored in 10 ml syringes. Before the scan, the specimens were inverted, a 1 ml sample was withdrawn for a blood gas analysis (Radiometer-ABL780, Radiometer, Brønshøj, Denmark), and then the samples were placed in the MRI scanner. A heart rate of 60/min was simulated on the identical 3 T scanner with the same OS-sequence as described in the imaging protocol for the in-vivo part of this study.

### In-vivo study

This study protocol was approved by the local research ethics board of the Institut de Cardiologie de Montréal (ICM #13-1447). In order to examine the relevance of hemodilution to clinical studies, 21 healthy volunteers were recruited to participate in this study by public advertisement. Inclusion criteria were age >18 years and ability to give informed consent. Participants with any history of cardiovascular, respiratory, cerebral or renal disease, pregnancy, smoking within the last 6 months or any contraindication for MRI scans were excluded. The subjects were required to refrain from the consumption of caffeinated beverages 12 h prior to the scan.

### Experimental protocol

An 18-Gauge i.v. line was placed in a cubital or ante-cubital vein for the initial measurement of the baseline hematocrit and hemoglobin concentration. After acquisition of baseline images, 1 L of Ringer’s Lactate solution was rapidly infused over 5–8 min with a pressure bag (300 mmHg). Immediately after rapid i.v. hydration, a second blood sample was acquired before a second set of images was acquired (hyper-hydration) using the identical protocol as that used for baseline scans. Non-invasive blood pressure was measured before and after hemodilution, heart rate was continuously recorded during the scans and rate-pressure product was calculated by multiplying the systolic blood pressure with the heart rate. A vasoactive breathing maneuver according to Fischer et al. was employed as an activation-dependent stimulus analogue to fMRI scans at baseline and after hemodilution [11].

### Imaging protocol

Images were acquired in a clinical 3 T MRI system (Magnetom Skyra, Siemens Healthcare, Erlangen, Germany) using a 30-channel coil setup with body and spine coils. For the in-vitro study, the samples were scanned with a simulated 60/min heart rate immediately after localization, after being placed in the scanner to minimize sedimentation. For healthy volunteers, all images were acquired during a breath-hold at end-expiration. Left-ventricular (LV) function was assessed using a standard ECG-gated balanced steady-state free-precession (bSSFP) sequence in six LV-centered radial long-axis slices (slice thickness: 8 mm; TR: 3.26; TE 1.43 ms).

One mid-ventricular slice was acquired during all oxygenation-sensitive scans using a retrospective ECG-gated bSSFP prototype sequence (slice thickness 10.0 mm, TR 3.49 ms, TE 1.57 ms, flip angle 35°). After acquisition of baseline images for both the normo- and hypervolemic state during a short breath-hold, the subjects performed 60 s hyperventilation (35 breaths/min) with constant cine acquisition during a maximal consecutive breath-hold as a dynamic vasoactive maneuver at both levels. A metronome was used to ensure consistency of the respiratory rate. Volunteers were instructed to use the alarm bell to signal the break-point of the voluntary apnea and to immediately recommence breathing.

### Image analysis

Image analysis was performed using certified CMR evaluation software (cvi42, Circle CVI, Calgary, Alberta, Canada). In the in-vitro samples, SI was measured after defining a contour in the long-axis (LAX) orientation of the syringes. For the assessment of left-ventricular function, parameters in the volunteers endo- and epicardial contours were traced in end-systole and end-diastole.

In OS-CMR images, manual endo- and epicardial contour tracing was completed in end-systolic frames. Mean myocardial SI was automatically generated by the software following contour tracing. All SI values were expressed as percent change (ΔSI[%]) between two images.

$$ \varDelta SI\ \left(\%\right) = \frac{SI(maneuver)-SI(baseline)}{SI(baseline)}*100 $$

The change in signal intensity between the baseline and hyper-hydration images was calculated, and the relative SI changes during the vasoactive breathing maneuvers were compared between the two hydration states. Specifically, differences between end-hyperventilation (HV), peak (SI_max_), as well as after 30 s apnea (SI_30s_) and end-breath-hold SI (SI_end_) changes were examined. A second reader, blinded to the hydration status, the maneuver and the subjects’ demographics, read 39 out of 220 images (18 %) to assess inter-observer reproducibility. For the in-vitro samples, the same equation was used to calculate the %change in SI from the undiluted blood samples (100 %) to the LRS-diluted samples (90, 80, 70, 60 and 50 %), respectively.

### Statistical analysis

Statistical analysis was performed using Prism 6 (GraphPad Software Inc., California, USA). All variables were checked for normal distribution using the D’Agostino-Pearson omnibus normality test. Student’s t tests were performed for those values with normal distribution, otherwise Mann-Whitney or Wilcoxon tests were used. To test correlation between quantitative variables, Pearson correlation coefficients or Spearman rank test were determined. Differences in blood levels to baseline in the in-vitro data was analyzed with a one-way ANOVA (Friedman test) with a Dunn post-hoc test for multiple comparisons. Variables are presented as means ± standard deviation. Images of nine participants (39 readings) were replicated by a second independent reader, and inter‑observer reliability was assessed using a two-way intraclass correlation (ICC) test with SPSS version 23 (SPSS IBM, New York, USA). *P* < 0.05 was deemed significant.

## Results

### In-vitro data

The changes in hemoglobin concentration, hemoglobin saturation, absolute deoxy-hemoglobin (deoxyHb) concentration and the deoxyHb fraction of arterial and venous blood samples are shown in Table 1. Values differed significantly between the undiluted (A100 and V100) and the diluted samples (*p* < 0.001). Arterial blood had no detectable deoxyHb. In the venous blood, the absolute deoxyHb concentration (*p* < 0.001) and the fraction of deoxy-Hb fell with increasing dilution (*p* < 0.001), while oxygen saturation showed a small increase (*p* < 0.001). Actual hemoglobin concentration for the same dilution of venous and arterial samples did not differ.

**Table 1:** Changes in blood parameters during hemodilution of arterial and venous blood of healthy swine in vitro

	Total Hb Conc. (g/L)	FHHb (%)	DeoxyHb Conc. (g/L)	SO_2_ (%)
A100	92.0 ± 6.3			
A90	81.0 ± 5.8*			
A80	75.2 ± 12.3*			
A70	65.0 ± 10.7*			
A60	60.6 ± 6.1*			
A50	45.8 ± 7.12*			
V100	93.8 ± 7.0	34.7 ± 10.6	32.7 ± 11.4	64.3 ± 10.9
V90	87.2 ± 9.0*	33.8 ± 8.0*	29.4 ± 7.1*	65.2 ± 8.1*
V80	79.0 ± 9.6*	32.7 ± 9.2*	25.8 ± 7.5*	66.2 ± 9.4*
V70	72.2 ± 12.7*	31.4 ± 8.9*	22.5 ± 6.5*	67.6 ± 9.1*
V60	68.8 ± 12.0*	29.3 ± 9.8*	20.9 ± 10.0*	69.7 ± 10.0*
V50	47.2 ± 5.2*	26.4 ± 9.6*	12.2 ± 3.3*	72.7 ± 10.0*

Changes of blood parameters: There is a significant decrease in hemoglobin (Hb), deoxyHb fraction (FHHb) and absolute concentration, while oxygen saturation increased compared to baseline with increasing dilution steps. *P < 0.001 for changes in blood parameters of the diluted samples compared to the undiluted arterial (A100) or venous (V100) baseline

**Fig. 1:**
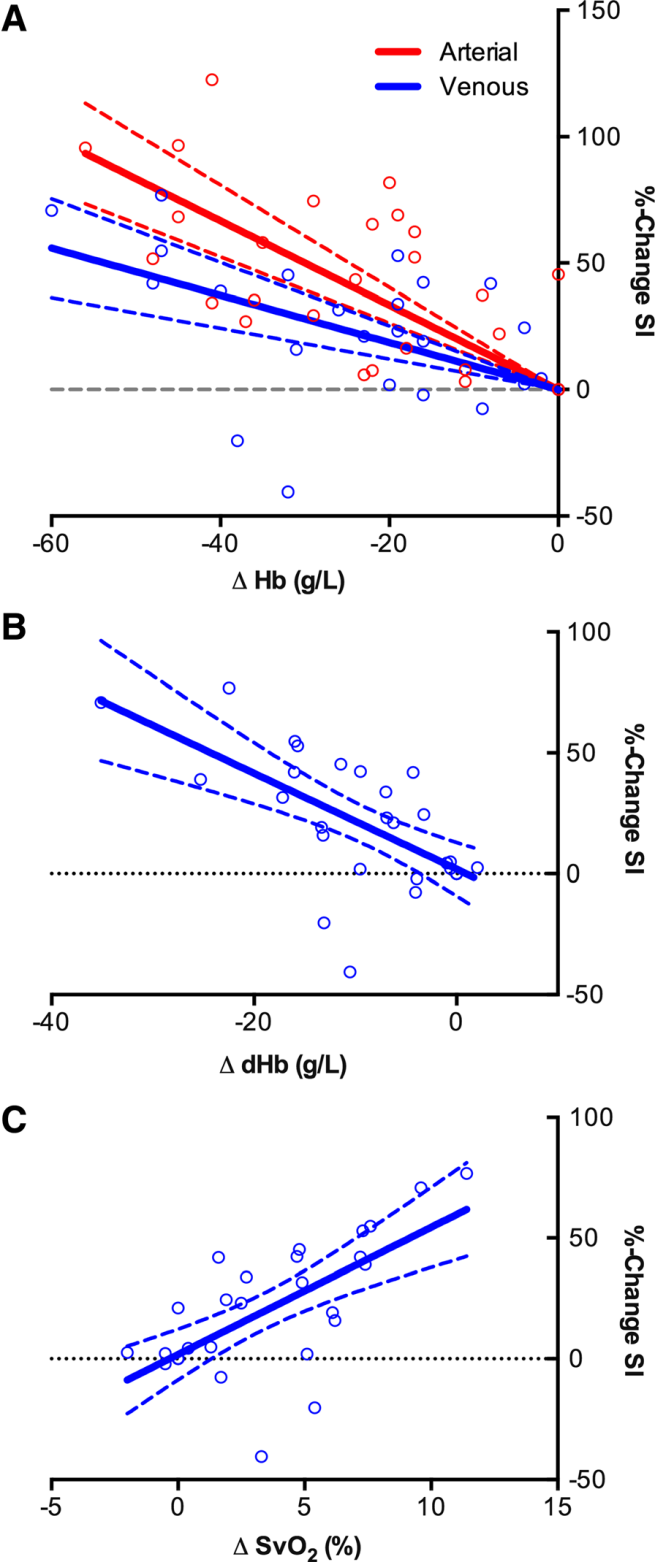
Relationship between signal intensity changes with blood results. **a** Relationship between the differences in hemoglobin concentration of the blood samples compared to baseline with changes in oxygenation-sensitive signal intensity of arterial and venous blood samples in vitro. Differences in hemoglobin concentration were negatively correlated to changes in signal intensity for venous (*r* = -0.55, *p* = 0.002) and arterial blood (*r* = -0.67, *p* < 0.0001). **b** Differences between absolute deoxyhemoglobin concentration (g/L) in venous blood and SI changes (%). There was a moderate relationship between the difference in hemoglobin concentrations and changes in SI (-0.65, *p* < 0.0001). **c** Relationship between changes in hemoglobin saturation of venous blood (SvO2) and changes in oxygenation-sensitive signal intensity (%change SI). There was a moderate correlation between venous hemoglobin saturation and signal intensity changes (*r* = 0.68, *p* < 0.0001)

The % change in SI from the baseline sample for venous (*r* = -0.55, *p* = 0.002) and arterial blood (*r* = -0.67, *p* < 0.0001) were negatively correlated with the changes in hemoglobin concentration, respectively (Fig. 1a). Further, there was a relationship between changes in SI and SO_2_ (*r* = 0.68, *p* < 0.0001, Fig. 1b) as well as the deoxyHb concentration of venous blood (-0.65, *p* < 0.0001, Fig. 1c).

### In-vivo data

#### Demographics

The participants’ demographics are displayed in Table 2. While there was no difference in age and body mass index (BMI), height (*p* < 0.001) and weight (*p* < 0.001) were significantly higher in males. All variables passed the D’Agostini-Pearson Test for normal data distribution.

**Table 2:** Participant demographics

Parameter	All (*n* = 21)	Male (*n* = 11)	Female (*n* = 10)	*p*-value
Age (years)	21.4 ± 6.7	29.0 ± 1.8	28.0 ± 2.4	*p* = 0.69
Height (cm)	168.7 ± 10.5	175.8 ± 2.0	160.8 ± 2.6	* p **< 0.001**
Weight (kg)	67.6 ± 14.1	76.6 ± 3.1	57.8 ± 3.4	* p **< 0.001**
BMI (kg/m^2^)	23.6 ± 3.3	24.5 ± 0.8	22.3 ± 1.1	*p* = 0.09

Participant demographics: There was a difference in height and weight between male and female participants (*p < 0.001), however, BMI and age did not differ

#### Hemoglobin concentration and hematocrit

Rapid infusion of 1 L Lactated Ringer’s solution resulted in a significant drop in the hemoglobin concentration (142.5 ± 15.2 g/L vs. 128.8 ± 15.2 g/L; *p* < 0.0001) and hematocrit (42.0 ± 4.1 % vs. 38.2 ± 4.0 %, *p* < 0.0001). There was no difference in relative hemoglobin (-12.73 ± 2.1 vs. -12.6 ± 2.4 g/L, *p* = 0.5) or hematocrit (-4.4 ± 0.6 vs. -3.2 ± 0.7 %, *p* = 0.2) changes between male and female participants.

#### Breath-hold performance

Maximal voluntary apnea was shorter after hemodilution (68.4 ± 20.1 s vs. 79.6 ± 21.2 s; *p* = 0.001). There was a weak but significant correlation between hemoglobin concentration and breath-hold time (*R* = 0.31, *p* = 0.0495)

#### Left-ventricular function parameters

There was a significant increase in end-diastolic volume, stroke volume, cardiac output and the rate pressure product during hemodilution (Table 3).

**Table 3:** Changes in left-ventricular function parameters

Cardiac parameter	Normovolemia	Hypervolemia	*p*-value
End-Diastolic Volume (mL)	142.5 ± 8.0	147.3 ± 7.9	* p **< 0.01**
End-Systolic Volume (mL)	53.5 ± 4.1	53.5 ± 4.2	*p* = 0.998
Stroke Volume (mL)	89.0 ± 4.7	93.7 ± 4.3	* p **< 0.005**
Ejection Fraction (%)	63.1 ± 1.3	64.3 ± 1.3	*p* = 0.065
Cardiac Output (mL)	5710 ± 330	6177 ± 323	* p **< 0.005**
HR (beats/min)	64.3 ± 1.7	65.8 ± 1.6	*p* = 0.167
Rate-Pressure Product	8012 ± 366	8733 ± 345	* p **< 0.001**
Systolic Myocardial Mass (g)	115.8 ± 7.2	114.4 ± 6.9	*p* = 0.114

Left-ventricular function parameters: After rapid infusion of 1L Lactated Ringer’s solution. There was an increase in end-diastolic volume, stroke volume, cardiac output and rate-pressure product after hyperhydration (*p < 0.01)

#### Image quality

In oxygenation-sensitives images 18 out of 680 (2.65 %) end-systolic image frames had to be excluded due to poor image quality mostly due to breathing artifact at the transition from hyperventilation to apnea. In all other images image quality was good to excellent. There was no difference in image quality between the hydration states (2.47 % vs. 2.86 % exclusion rate). A strong intra-class correlation coefficient (ICC) of 0.924 (95 % CI: 0.0801–0.969, *p* < 0.001) indicated a strong agreement between readers.

#### Influence of hemoglobin concentration on oxygenation-sensitive signal intensity changes

**Fig. 2:**
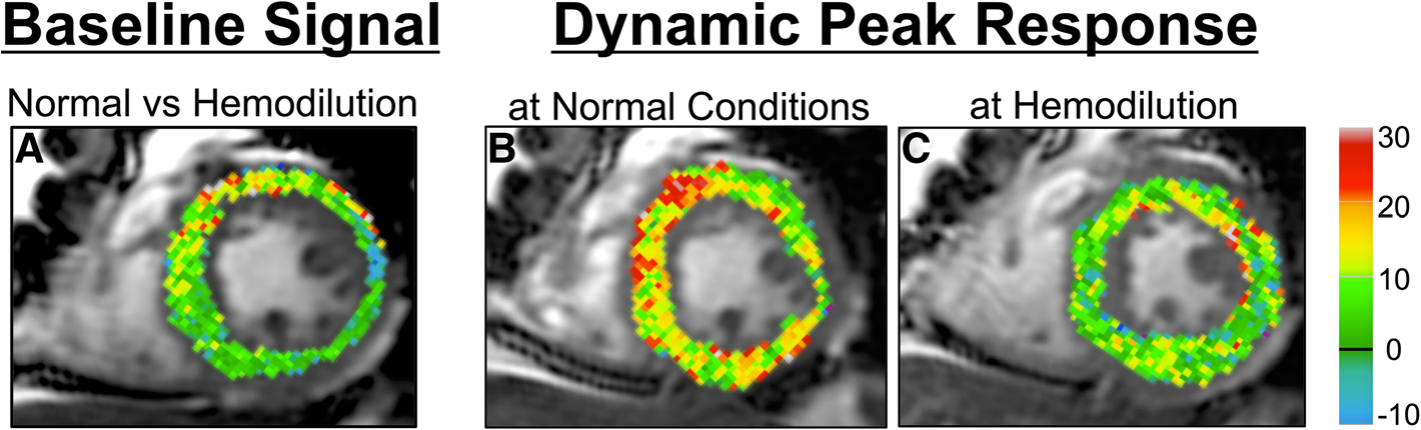
Differences in signal intensity changes between hydration states. DICOM subtraction images demonstrate that hemodilution increases the OS signal at rest (**a**). Furthermore, the breathing maneuver could significantly induce a transient increase in myocardial oxygenation in normal conditions (**b**), but this response was attenuated in a hemodiluted state (**c**)

Baseline myocardial SI in OS-CMR images increased by 3.0 ± 5.7 % (*p* = 0.026) following rapid infusion. Figure 2a shows the increase in baseline SI in a subject after hemodilution at rest. There was no relationship observed between hemoglobin concentration and changes in SI in the entire collective (*p* = 0.56) or in females (*p* = 0.19), however in the eleven males there was a strong association between absolute changes in hemoglobin concentration and %changes in SI (*R* = 0.82, *p* = 0.002, Fig. 4a).

**Fig. 3:**
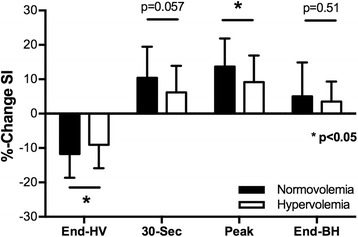
Differences in signal intensity changes between hydration states. SI changes during vasoactive breathing maneuvers and after hyperventilation (End-HV), peak values and values at the end of a maximal breath-hold (End-BH) during normovolemia and hypervolemia in healthy subjects (*p* < 0.05)

SI decreased after hyperventilation and increased during apnea at both hydration states. However, an attenuation of signal intensity response to the breathing maneuvers was observed after hyperventilation (HV, *p* = 0.037) and at the maximal SI increase during apnea (SI_max_, *p* = 0.012) of the breathing maneuver. At 30 s (SI_30s_) into the breath-hold, there was only a trend (*p* = 0.057) for an attenuated SI increase at hyperhydration (Fig. 3). Although breath-hold length was significantly shorter after rapid infusion, there was no difference for the time point of maximal signal intensity (SI_max_) during apnea (34.03 ± 13.09 vs. 29.51 ± 11.59 s, *p* = 0.18).

#### Relationship between hemoglobin changes and SI attenuation during vasoactive breathing maneuvers

**Fig. 4:**
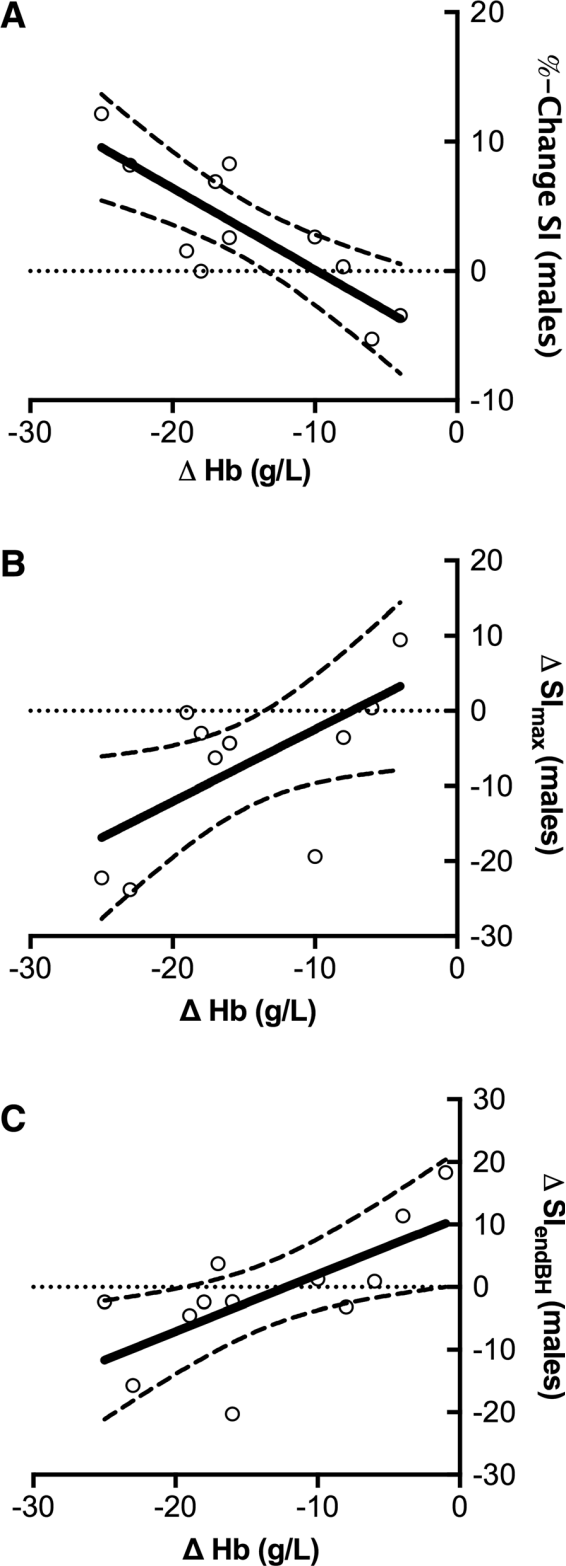
Relationship of baseline oxygenation-sensitive signal intensity and signal intensity response in relationship to hemoglobin changes in healthy males. **a** There was strong negative relationship between changes in hemoglobin concentration and signal intensity on oxygenation-sensitive images before and after rapid infusion of Lactated Ringer’s solution in healthy men (*r* = 0.82, *p* = 0.002) at rest, suggesting an increase in relative SI with decreasing hematocrit. **b** Relationship between the extent of hemodilution and the attenuation of the maximal signal intensity response during apnea (*r* = 0.63, *p* = 0.037) and **c**) at the end of the breath-hold (*r* = 0.68, *p* = 0.016) in healthy males. A significantly decreased hemoglobin level was associated with a stronger attenuation of the signal intensity response in healthy males

With increasing reduction in hemoglobin concentration (∆Hb), we found a stronger attenuation in SI (difference in SI-response in identical maneuver) for SI_max_ in males (*r* = 0.63, *p* = 0.037) but not in females (*r* = 0.24, *p* = 0.51), resulting in only a trend for a correlation for the entire cohort (*r* = 0.42, *p* = 0.058), (Fig. 4b). Figure 2b  shows the difference in the SImax in one subject before and after hemodilution. SI attenuation was also found to be correlated to the changes in hemoglobin concentration at the end of the breath-hold (SI_end_) in the entire collective (*r* = 0.55, *p* = 0.012). Looking at gender differences this was also true for the male subgroup (*r* = 0.68, *p* = 0.016, Fig. 4c) but did not apply in females (*r* = 0.54, *p* = 0.105).

## Discussion

Our results demonstrate for the first time that hemodilution may increase baseline signal intensity in oxygenation-sensitive images but also attenuate the observed signal response to vasoactive breathing maneuvers. The understanding of this relationship is important for the clinical applications of OS-CMR [2]. Patients with changing fluid status may show altered signal intensity responses, which may confound results and their interpretation. During heart failure, for example, an attenuated signal intensity response may underestimate coronary vascular reactivity or even falsely suggest inducible oxygenation deficits.

In the in-vitro data, the concentration and fraction of deoxyHb were negatively correlated to the signal intensity of the sample and positively correlated to the hemoglobin saturation. Interestingly, both the hemodilution of arterial and venous blood resulted in an increase in signal intensity of the sample. Although there was no significant difference in the slope of the regressions, the less pronounced increase observed in venous blood can be explained by the signal-attenuating effects of the deoxyHb.

During the dilution of deoxyHb, the T2* effect based on the paramagnetic properties of deoxyHb is reduced, which augments the relative impact of T2 effects, leading to a higher signal intensity. Establishing this relationship in vitro is an important step to distinguish the SI effects of the simple hemodilution of deoxyhemoglobin from the more complex SI-changing effects by the vasoactive breathing maneuver in the participants during hemodilution.

The in-vitro data corresponds to the increase in baseline signal intensity after rapid infusion of crystalloids in healthy volunteers, leading to an attenuation of the T2* effect, which explains the observed rise in signal intensity. This effect was also observed in neurologic fMRI studies by Lin et al. [17, 18]. The group assessed the effect of mild and moderate acute hemodilution. The group concluded that intra-voxel concentration of deoxyhemoglobin was the main contributing factor causing changes in signal intensity. It was shown that the increase in signal intensity (or reduction in R2* or inverse of T2*) was proportional to the decrease in hematocrit.

The linearity of the regression lines in our results suggests that the baseline signal intensity can be normalized for a known deviation of hemoglobin concentration. Interestingly, the change in hemoglobin concentration was only related to changes in signal intensity in male subjects.

Levin et al. (2001) performed repeated photic-stimulation functional MR experiments before and after hemodilution [19] in male subjects. Corresponding to a 6 % decrease in hematocrit, the authors observed a statistically significant reduction of the inducible response of 8 %. These results are comparable to our data. Interestingly, in our study the extent in signal intensity attenuation was only correlated to the increase of hemodilution in males and not in females, which showed a preserved response, despite a similar level of hemodilution, similar body mass index values and the same infusion volume. From the present data, we cannot be sure about the physiologic reasons for the absence of such a relationship in females. Yang et al. found that the relationship between BOLD-SI changes in the brain and hematocrit levels are modulated by sex [20]. In their study, the inter-hemispheric connectivity within the pars occularis extending to the precentral gyrus was found to be positively correlated with hematocrit in females and negatively correlated in males. However, the study could not clarify if these affects were acute or to neural adaptations to chronic hematocrit differences.

Yet, the observed sex differences in our study may be explained by a higher total vasodilatory capacity which had not been exhausted by the intravascular hypervolemia. A larger sample size might have detected a weaker yet still existing relationship in female participants.

The increase in baseline SI and the attenuation in SI response during vasoactive breathing maneuvers were already observed at a mean drop in hemoglobin concentration of 13.7 g/L (corresponding drop in HCT 3.8 %) in our study. Such a relatively small change is easily encountered in clinical settings. In some patients, the drop in hemoglobin concentration during volume substitution after blood loss can exceed such change by far. Conversely, diuretic therapy may significantly increase in hematocrit level. Thus, our data have clinical relevance for the future use of OS-CMR in non-invasive cardiac testing.

The changes in ventricular volumes and function data are explained by the increase in preload and consecutive improvement in contractility, based on the Frank-Starling mechanism. The increase in blood pressure leads to an elevated rate-pressure product, with an increased myocardial workload and thus higher oxygen extraction. While this may suggest an explanation for the decreased signal intensity response as well, the blood pressure remained within the auto-regulation range for myocardial blood flow and therefore, a decreased myocardial oxygen supply would result in a consecutive compensatory increase in blood flow to maintain a constant tissue oxygenation. Thus, an increase of the myocardial oxygen extraction less likely explains the observed hemoglobin-dependent changes in OS-CMR.

A study of Zheng and colleagues on brain functional connectivity in hemodialysis patient with end-stage renal disease [22] showed that a weakening of the cortical and subcortical network connectivity in these patients was related to anemia. This observation could be explained by a decreased T2* effect in anemic patients. Another study observed a blunted myocardial oxygenation response to adenosine in patients with chronic kidney disease and renal transplant patients [13]. The authors attributed this finding to the possible presence of microvessel disease, as these patients have a higher incidence of cardiovascular events. However, the authors do not state if the MRI scans were performed before or after dialysis in these patients. The differences in fluid state before and after dialysis could have had a profound effect on the measured OS-signal response. It is possible that the SI response may have been underestimated, which leads to the conclusion of the presence of coronary artery or microvascular coronary disease in these patients. Further studies are warranted to assess the relationship between altered fluid status, OS-signal response and heart disease in order to deduct correct conclusions.

### Limitations

The precise execution of breathing maneuvers may have varied between participants and between breathing maneuvers within subjects. To minimize variations, we paced the respiratory rate for the subjects with a metronome during hyperventilation and visually monitored it by a camera installed in the scanner room. If breathing appeared insufficiently deep or too slow, we immediately intervened to correct that. As breath-hold duration during maximal tolerable apnea is highly variable, we used standard time points for comparison between fluid states. The 30 s time point was reached by every participant. Further, we used the peak signal intensity change during apnea and the signal intensity change at the end of the maneuver, when participants had the urge to breathe again for comparison. However, these time points vary in the time point of acquisition during the maneuver between participants and between runs. The primary reader of the exams was not blinded to the fluid state during which the images were acquired. However, the second reader, who analyzed 18 % of the images, was blinded to the fluid state, and there was an excellent agreement between the readers. Results may be very different in a patient cohort with a more chronic systemic fluid overload. Although our data opens up the potential for normalizing the measured signal intensity response to hemoglobin changes, there needs to be further research, especially in patients with coronary pathologies present in patients with heart failure that can be scanned before and after re-compensation. Larger sample sizes may allow for a more precise determination of the relationships and thus correction factors.

## Conclusion

In dynamic studies using oxygenation-sensitive CMR, the hematocrit level affects baseline signal intensity and the observed signal intensity response. Thus, the hydration status of the patient may be a confounder for OS-CMR image analysis.

## Abbreviations

∆, delta (difference); ∆OS-SI_max_, delta (difference) in maximal oxygenation-sensitive signal intensity; A, arterial blood sample; ANOVA, analysis of variance; BH, breath-hold; BMI, Body Mass Index; BOLD, Blood Oxygen Level-Dependent; bSSFP, balanced steady-state free-precession; CI, confidence interval; cm, centimeter(s); CMR, Cardiovascular Magnetic Resonance; Conc., concentration; CT, Computed Tomography; deoxyHb, deoxygenated hemoglobin; ECG, electrocardiogram; FHHb, fraction of deoxyhemoglobin of total hemoglobin; fMRI, Functional Magnetic Resonance Imaging; g, gram(s); h, hours; Hb, hemoglobin; HCT, hematocrit; HR, heart rate; HV, hyperventilation; i.v., intra-venous; ICC, intra-class correlation; ICM, Institut de Cardiologie de Montréal; kg, kilogram(s); L, Liter; LAX, long-axis; LRS, Lactated Ringer’s solution; LV, left-ventricular; m, meter(s); ml, milliliter(s); mmHg, Millimeter Mercury; MR, Magnetic Resonance; MRI, Magnetic Resonance Imaging; OS, oxygenation-sensitive; s, seconds; SAX, short-axis; SI, signal intensity; SI_30s_, signal intensity at 30 s into the breath-hold; SI_end_, signal intensity at the end of the breath-hold; SI_max_, maximal signal intensity during breath-hold; SO_2_, oxygen saturation of hemoglobin; SvO_2_, venous oxygen saturation of hemoglobin; T, Tesla; TE, Echo time; TR, Repetition time; V, venous blood sample; vs., versus
